# Prediction of Selected Mechanical Properties of Polymer Composites with Alumina Modifiers

**DOI:** 10.3390/ma15030882

**Published:** 2022-01-24

**Authors:** Ewelina Kosicka, Aneta Krzyzak, Mateusz Dorobek, Marek Borowiec

**Affiliations:** 1 Department of Production Engineering, Lublin University of Technology, Nadbystrzycka 36, 20-618 Lublin, Poland; 2 Department of Airframe and Engine, Military University of Aviation, Dywizjonu 303 no. 25, 08-521 Dęblin, Poland; a.krzyzak@law.mil.pl; 3 Faculty of Electronics and Information Technology, Warsaw University of Technology, Nowomiejska 15/19, 00-665 Warsaw, Poland; dorobekmateusz@gmail.com; 4 Department of Applied Mechanics, Lublin University of Technology, Nadbystrzycka 36, 20-618 Lublin, Poland; m.borowiec@pollub.pl

**Keywords:** neural networks, modeling, composites, machine learning, L-BFGS

## Abstract

Forecasting is one of the cognitive methods based on empirical knowledge supported by appropriate modeling methods that give information about the way the relations between factors and how the phenomenon under study will develop in the future. In this article, a selection is made of a suitable architecture for a predictive model for a set of data obtained during testing of the properties of polymer composites with a matrix in the form of epoxy resin with trade name L285 (Havel Composites) with H285 MGS hardener (Havel Composites), and with the addition of the physical modifier noble alumina with mass percentages of 5%, 10%, 15%, 20% and 25% for the following grain sizes: F220, F240, F280, F320, F360, respectively. In order to select the optimal architecture for the predictive model, the results of the study were tested on five types of predictive model architectures results were tested on five types of prediction model architectures, with five-fold validation, including the mean square error (MSE) metric and R2 determined for Young’s modulus (E_t_), maximum stress (σ_m_), maximum strain (ε_m_) and Shore D hardness (⁰Sh). Based on the values from the forecasts and the values from the empirical studies, it was found that in 63 cases the forecast should be considered very accurate (this represents 63% of the forecasts that were compared with the experimental results), while 15 forecasts can be described as accurate (15% of the forecasts that were compared with the experimental results). In 20 cases, the MPE value indicated the classification of the forecast as acceptable. As can be seen, only for two forecasts the MPE error takes values classifying them to unacceptable forecasts (2% of forecasts generated for verifiable cases based on experimental results).

## 1. Introduction

Artificial intelligence (AI) is a solution that has unquestionably revolutionized many areas of the economy, as evidenced by the observed intensification of its applications. Previously identified exclusively with IT areas of activity, together with progressive digitization and computerization, it has turned out to be a human support tool in many unprecedented applications [1]. Its increasingly bold use provides an incentive to verify the potential for improvement in many areas, especially in the context of the ability to make inferences from Big Data [2]. Learning about and predicting processes or properties based on AI is thus becoming the basis for informed object management, optimization of final properties or incurred costs, or effective risk management policy [3,4]. Taking these factors into account, it is important to be aware of the benefits obtained not only in financial terms, but also in respect of safety and ecology.

The analysis of the literature shows that in engineering science is more and more observing use of artificial intelligence in forecasting of certain properties or dependencies. However, there is no found description of the prediction of mechanical properties of composites in the increasingly popular Phyton programming language. The empirical research on the mechanical properties of polymer composites with alumina allowed for the construction of the database and this is discussed in this paper. The set collected in this way was applied to use Phyton in predicting the properties of polymer composites with alumina.

### The Role of AI in Engineering Issues

Work on innovative engineering solutions or implementations that improve processes or increase efficiency generally accepts alternative support tools, as long as they show prospects for success [5,6,7,8,9,10,11]. The application of AI in engineering solutions yielding the desired results has encouraged numerous research works presenting a variety of applications. Artificial intelligence has emerged, among others, in the context of monitoring or modelling the operation of machines or equipment [12,13], development and evaluation of manufacturing technologies [14,15,16,17], new engineering materials [18,19] or as support for civil engineering [20,21], as well as transport [22], electrical [23] or geological engineering [24]. It is impossible to mention all the areas of engineering problems in which it is applied, and the literature presented only signals the openness of the research community to its implementation.

The aforementioned possible areas of application of AI in the development of new engineering materials gives a perspective to build on the existing research results and model the properties of new materials subjected to modifications. The interest in applying just AI within material improvements is primarily due to the ability to describe the obtained features shaped in a non-linear manner. Thus, for example, in [25], the authors proposed a machine learning approach to predict the effective elastic properties of composites with arbitrary shapes and inclusion distributions. They developed a method of spline neural networks to predict the effective Young’s modulus and Poisson’s ratio of composites. Through numerical experiments, they demonstrated that a trained network can effectively provide an accurate representation between effective mechanical properties and microstructures of composites with complex structures. In contrast, Chen et al. [26] investigated an integrated surrogate approach based on direct finite volume averaging micromechanics (FVDAM) and long short-term memory (LSTM) to predict the elastoplastic response of composite materials. The structure proposed in this article can provide a viable alternative for determining the history-dependent response of composites directly from data analysis without the need to understand the underlying deformation mechanism in homogenization techniques, and according to the authors, provides a basis for efficient multi-scale analysis of composite materials and structures.

Reference [27] presents the basic structure of backpropagation (BP) and radial basis function (RBF) neural networks for developing predictive models to study the properties of Pb-Al composites. The model verification results showed that neural networks can present the trend of shear stress variation, which is an indicator of physical properties. Two types of networks were used to establish a predictive model to study the properties of Pb-Al composite material. By comparing their learning speed in the process of experimentation and checking the prediction results, it is found that an RBF neural network is better than a BP neural network in property prediction and its validation results show better trend of variation of prediction rate. An RBF neural network can have a wide range of use to study the properties of Pb-Al composites in the future.

In [28] a three-layer artificial neural network (ANN) model with feedback was developed to predict the compressive strength of layered E-glass/polyester composites processed using the VARTM method. The composites were made from fabric preforms combined with 0, 3 and 6 wt% thermoplastic binder. The learning of the artificial neural network was done using a back propagation algorithm. Good agreement between measured and predicted values was obtained. The authors of this paper concluded that the ANN model yields better predictions than the MLR model for experimental data and that the ANN model can achieve the desired level of compressive strength values at lay-up for composites processed with the addition of a thermoplastic binder.

In turn, [29] determined the mechanical properties of glass fibre reinforced composite cut laminates made by transfer moulding resin at different injection pressures based on an experimental design. A learning-based optimization (TLBO) to predict the maximum mechanical properties of the composite by selecting the number of layers and injection pressure was proposed. An artificial neural network (ANN) with a feedback propagation algorithm was also used to predict responses and compare them with experimental results.

In [30], the authors developed an extreme learning machine (ELM) model, which is a state-of-the-art data analysis-based model, for fatigue cycle prediction of composite materials for modelling the mechanical behaviour of fibre-reinforced composite materials. Predictions were created from specific experimental data, determining the number of cycles based on predetermined loading conditions. The authors additionally considered several input variables for modelling (geometry dimension, stress and orientations) and the results of the ELM modelling method were compared with an artificial intelligence-based model called a generalized regression neural network (GRNN). The results of the prediction test suggested better performance of the tested modern ELM model compared to the classical GRNN. Furthermore, the best attributes for running optimal predictive models were the geometry of the samples and the applied stresses.

The fatigue strength prediction was also applied by the Al-Assadi et al. [31]. In their research, they looked at testing different neural network architectures for prediction. Learning was performed on specific composites and predictions were made for different materials. The results showed that SSN can be used to predict the fatigue strength of composite material. It was also observed that a single architecture/training method combination does not always give the best results for all materials.

Paper [32] presents artificial neural network models for fatigue life evaluation of unidirectional glass fibre reinforced epoxy composites under tensile-tension and tensile-compression loads. The fatigue behaviour of composite materials was described by three parameters: fibre orientation angle, stress ratio and maximum stress. These parameters were the input vectors and the number of cycles corresponding to the failure was taken as the output parameter. The network architecture was selected based on detailed parametric studies. It was trained and tested based on analytically generated data using finite element analysis. The predicted results of the neural network model were compared with the available experimental values and found to be consistent.

In [33] the erosion of polyether ketone (PEK) reinforced with different contents (0–30 wt%) of short glass fibres was investigated. Steady-state erosion rates were evaluated at different angles (15–90°) and impact velocities (25–66 m/s) using quartz sand particles as erodents. Artificial neural networks were used here to predict erosion rates based on an experimentally measured database of PEK composites. The effect of different learning algorithms on the learning performance of neural networks was investigated. As the authors point out, the predicted erosion rates were in good agreement with the experimentally measured values, which they believe demonstrates the ability to analyse the dependence of erosive wear on material composition and test conditions using relatively small experimental databases.

On the other hand, Jiang et al. [34] addressed the application of artificial neural network to predict the mechanical properties and wear of short fibre reinforced polyamide (PA) composites. To train the neural network, they used two experimental databases: one consisted of 101 independent fretting wear tests of PA 4.6 composites, while the other database was from a commercial company (including impact, tensile and bending test results). As presented in their publication, the properties of these composites can be well predicted by optimized and trained neural networks, and the ANN technique can more efficiently use of relatively limited experimental databases, which means significant time and cost savings in both research and production.

The cited authors, as well as authors of other publications in the field of application of artificial intelligence in shaping new structural materials, openly point to the potential of using forecasting methods in the field of materials engineering. It is an indicator towards sustainable production of composites taking into account safety, financial as well as environmental aspects.

## 2. Materials and Method

**Figure 1:**
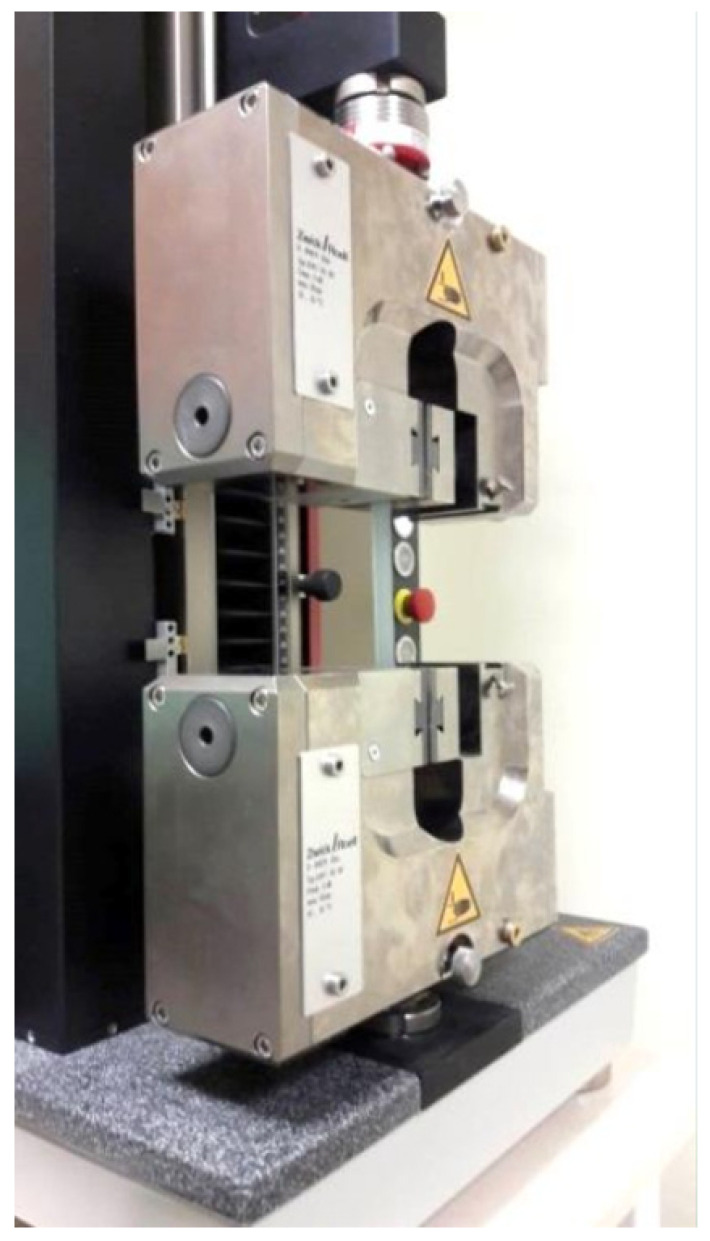
Zwick Z5.0 TN ZwickLine testing machine.

In this article, the results obtained in the determination of selected mechanical properties of composites with epoxy resin matrix, in which the reinforcing phase was noble alundum (symbol EA) Al_2_O_3_ of 99% purity and F220, F240, F280, F320, F360 grains (abrasive grains determined according to FEPA standard 42-2-2006 [35]). The results also include a reference series labelled F0 (without the addition of alundum). An epoxy resin with the trade name L285 was used to prepare the samples by gravity casting (Havel Composites, Svésedlice, Czech Republic) with H285 MGS hardener (Havel Composites), and noble alundum with mass percentages of 5%, 10%, 15%, 20%, and 25% for each of the listed grain sizes, respectively. Ultrasonic waves were used to remove air bubbles after forming the shape of the specimens while still in the liquid resin state. The number of each percentage of each series of samples differing in grain size of the alundum was set to 10. The specimens, in accordance with PN-EN ISO 527-2:2012 [36], were subject to a static tensile test using a Zwick Z5.0 TN ZwickLine testing machine shown in Figure 1, (ZwickRoell AG, Ulm, Germany).

**Figure 2:**
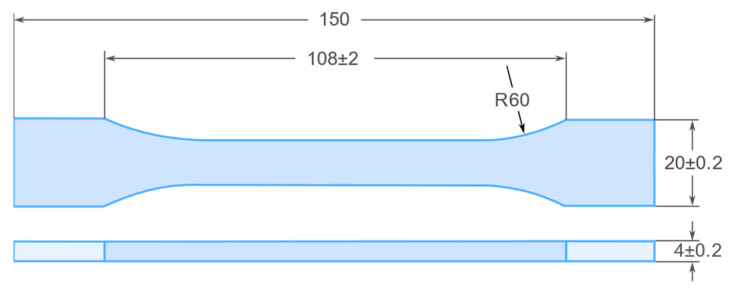
Dimensions of composite specimens for the tensile strength test (mm).

Tensile testing was performed with the following parameters: measuring length 90 mm, cross-head speed 2 mm/min. The dimensions of the specimens are shown in Figure 2.

**Figure 3:**
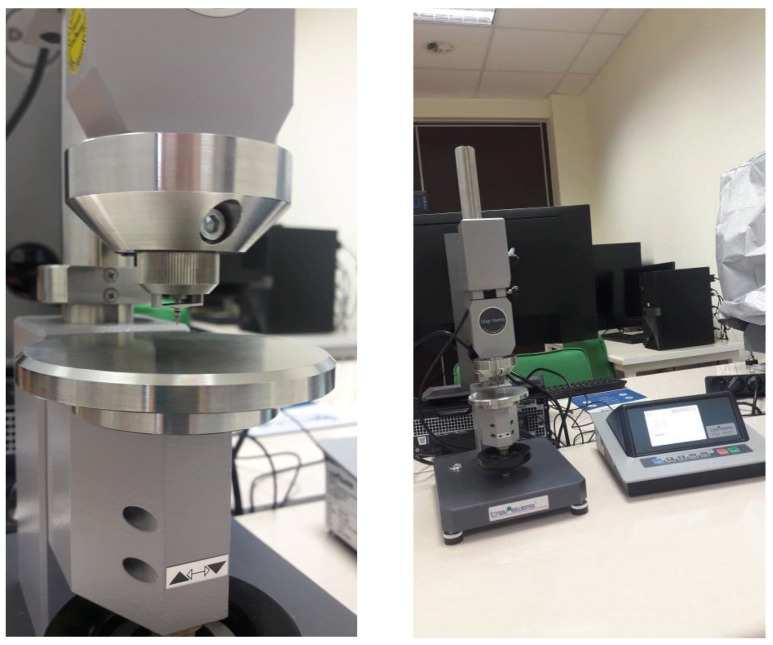
Bareiss Shore/IRHD Digi Test II, FRT GmbH, Bergisch Gladbach.

Apart from the static tensile test, hardness measurements were carried out using the Shore D method on a Bareiss digi test II hardness tester (Bareiss Shore/IRHD Digi Test II, FRT GmbH, Bergisch Gladbach, Germany, Figure 3) according to ISO 868 [37].

The research conducted to date made it possible to obtain the mechanical characteristics of polymer composites characterized by non-linearity, which causes problems with fitting many prediction models. The prepared datasets from the experimental studies formed the basis for data analysis and machine learning based on the Python 3.7 programming language. Several libraries—Pandas, NumPy, matplotlib, Scikit-learn—were relied upon to build a network that predicts the properties of materials of a particular composition. The five types of model architectures described in Table 1 are from scikit-learn modules.

The Pandas library is one of the most powerful data analysis packages in Python. It includes data structures and tools designed for data processing that make data cleansing and analysis in Python easier and faster. The Pandas library is often used in conjunction with other tools designed for numerical data processing, such as NumPy and SciPy, analytical libraries such as stats models and scikit-learn, and libraries designed for data visualization (such as matplotlib). The Pandas package is geared towards array processing and offers many functions that operate on arrays and allows processing of data without a loop for [38].

Numerical Python (NumPy) is an open source numerical library in the Python language. It includes multidimensional array and array data structures. It can be used to perform many mathematical operations on arrays, such as trigonometric, statistical and algebraic procedures. Panda objects rely heavily on NumPy objects.

The matplotlib library allows one to create static, animated and interactive visualizations in the Jupyter notebook space.

Scikit-learn is a free machine learning algorithm library written in Python and built on top of the SciPy module. The scikit-learn module provides developers with a number of algorithms from the field of supervised and unsupervised machine learning in the form of a consistent programming interface.

**Figure 4:**
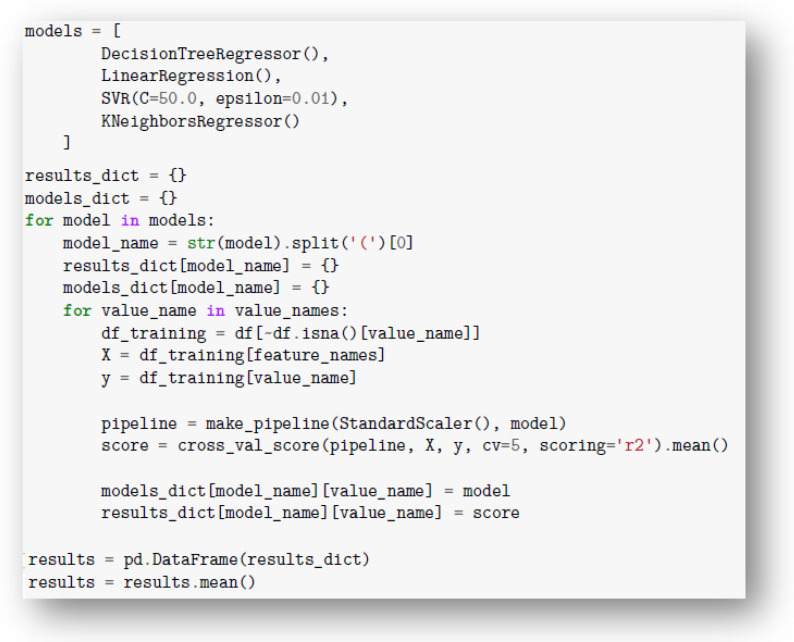
Program code snippet responsible for testing some predictive model architectures.

In order to select the optimal architecture for the predictive model, the results of the study were tested on five types of predictive model architectures results were tested on five types of prediction model architectures, with five-fold validation, including the mean square error (MSE) metric and R^2^ determined for the Young’s modulus (E_t_), maximum stress (σ_m_), maximum strain (ε_m_) and Shore D hardness (⁰Sh). The input data were standardized for training and prediction by the network. The program code fragment responsible for testing some types of architectures is shown in Figure 4.

The decision to choose the best forecasting architecture was based on the values of MSE (mean squared error), included in the price coefficients of ex-post forecast error. It is defined as:

(1) $$MSE=\frac{1}{n}\sum_{t=1}^{n}{\left(y_{t}-y_{t}^{P}\right)}^{2};\;\;\;\;\;t=1,2,3,\;\ldots ,\;n;$$

where $y_{t}^{P}$ represents the expired forecasts.

For the MSE results obtained, the smallest values are considered as the best. The preliminary results of the MSE values allowed us to conclude that the deep neural network (MLP Regressor) proves to be a better solution than the proposed decision trees, linear regression, SVR or K-nearest neighbours algorithm (Table 1).

Since MLP Regressor proves to be a better solution, the MLP Regressor results are discussed herein.

## 3. Results Discussion

The simulations tested variants of neural network models that differed in layer sizes and parameters. The one that minimizes the cost function was chosen. For this purpose, the coefficient of determination R^2^, which is one of the measures of the model fit to the learning data, was determined for each proposed network:

(2) $$R^{2}:=\frac{\sum_{i=1}^{n}{\left({\hat{y}}_{i}-\bar{y}\right)}^{2}}{\sum_{i=1}^{n}{\left(y_{i}-\bar{y}\right)}^{2}}\geq 0$$

where *y_i_*—i-th observation of variable *y*, $\hat{y}$*i_i_*—theoretical value of the explained variable (based on the model) and $\bar{y}$—the arithmetic mean of the empirical values of the explanatory variable.

Table 2 presents example results of the coefficient of determination for the simulations carried out for different network models. This table reported an information kind of activation functions that are functions of one-fold x from the previous layer or layers (logistic/identity), information about hidden layer sizes and the coefficient of determination R^2^ for each proposed network. The results are presented in order that R^2^ score from largest to smallest.

**Figure 5:**
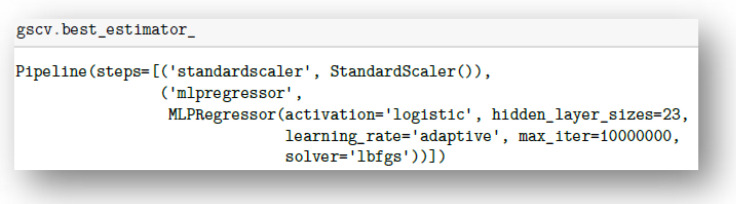
Code snippet generating parameters of the selected neural network.

On the basis of the obtained values of the coefficient of determination and the assumptions presented earlier, a network was selected which was characterized by the following parameters (Figure 5):

- Activation=‘logistic’;
- Hidden_layer_sizes;
- Learning_rate=‘adaptive’;
- Solver=‘Ibfgs’.

The given optimization algorithm from the Quasi-Newton family of methods, L-BFGS-B -limited-memory BFGS (BFGS with limited memory), is an algorithm for finding local extremes of functions that are based on Newton’s method of finding stationary points of functions. It uses Hessian inverse matrix estimation to control the search of the variable space. Unlike BFGS, which stores a dense approximation of the n x n inverse of the Hessian, L-BFGS stores only a few vectors that implicitly represent the approximation (saving memory). It is applied to optimization problems with a large number of variables and simple constraints [39].

The completion of the neural network training stage allowed us to move on to generating predictions. A part of this section resulted in the determination of the need to simulate the mechanical properties of composites for compositions that had not been empirically tested – the prediction was to include both additional, untested grain sizes, as well as untested mass percentages of individual alundum grain sizes (sampling from 1 to 25% in 1% increments). The generated prediction summary was exported to a spreadsheet. Due to also generating the values of selected mechanical properties for such polymer composites with the addition of alundum, which were subjected earlier to empirical investigations (grain sizes F220, F240, F280, F320, F360 and mass percentages 5%, 10%, 15%, 20%, 25%) it was possible to determine the values of prediction errors. ME (Mean Error) and MPE (Mean Percentage Error) were determined. Their values are presented in Table 3.

Based on objective criteria of acceptability of forecasts [40], according to which:

- Vτ ≤ 3%, the predictions are very accurate (Table 3 —green);
- 3% < Vτ ≤ 5%, we consider the predictions to be accurate (Table 3—blue);
- 5% < Vτ ≤ 10%, predictions may be acceptable (Table 3—yellow);
- Vτ >10%, predictions are unacceptable (Table 3—red);

appropriate colour coding of the forecasts has been applied, as described in brackets.

Analysing the above results, in 63 cases the forecast should be considered very accurate (green colour, this represents 63% of the forecasts that were compared with the experimental results), while 15 forecasts can be described as accurate (blue colour, 15% of the forecasts that were compared with the experimental results). In 20 cases (yellow), the MPE value indicated the forecast was to be classified as acceptable. As can be seen, only for two forecasts the MPE error takes values that classify them into unacceptable forecasts (red, 2% of forecasts generated for cases verifiable from experimental results).

**Figure 6:**
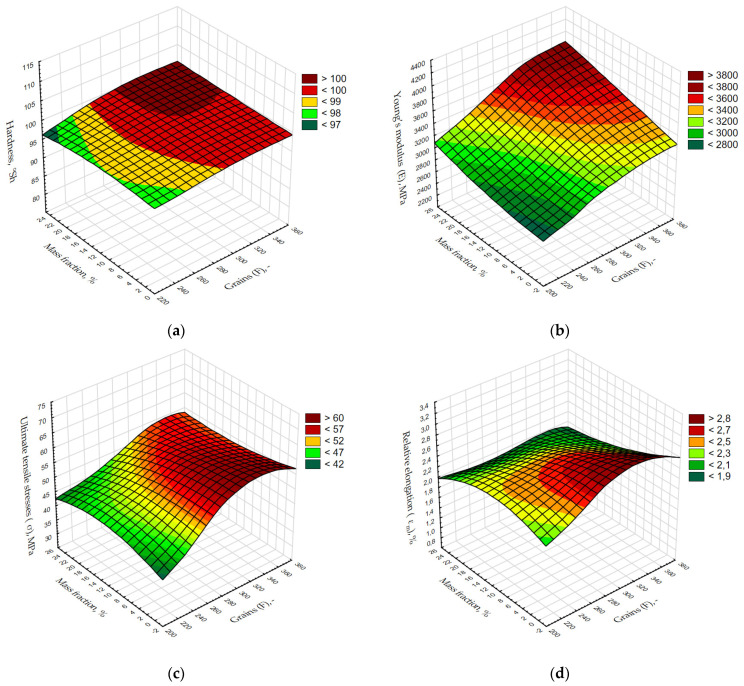
This 3D maps generated from the predicted values of hardness (**a**), Young’s modulus (E_t_) (**b**), ultimate tensile stress (σ_m_) (**c**), and relative elongation (ε_m_) (**d**).

The predictions obtained in this way give satisfactory results and encourage fragmentary experimental verification of new compositions of polymer powder composites for which only the values of mechanical properties generated by neural network are known. These in turn were determined for sampling from 1 to 25% with a step of 1% for grain sizes from F220 to F360 (with a step of 10) for simulated results of selected mechanical properties (Figure 6).

During laboratory tests, only a part of share modifiers at each grains of alumina was investigated. Figure 6 give a view on the consecutive mechanical properties of the investigated composites at such percentage of share modifiers (mass fraction) with its chosen grain sizes, which were not verified by experimental approach. When striving to manufacture a composite structure with increased hardness, it is advisable to apply the grain sizes F320, F340 and F360 at weight share in range 16–25% (Figure 6a). Similarly, in the case of the Young’s modulus, the highest of forecasted values include within 16–25% of weight mass modifier for grain size F300, F320, F340 and F360 (Figure 6b). Analysing forecasted values of maximum stress (σ_m_) and maximum strain (ε_m_) one can notice, that maxima are reached at grain size over F300, however the mass fraction in case of σ_m_ should reach up to 12% of alumina (Figure 6c), and for ε_m_ up to 6% (Figure 6d). The main purposed of the paper has been reached by discussed the way of verification of the forecasting algorithm possibilities to predict values of chosen mechanical properties of composite materials. It is useful approach which was not applied for such materials earlier. Based on experimental studies, it is known that analysed properties are nonlinear characteristics. Reported knowledge of the mechanical properties behaviour of polymer composites including a physical modifier will be a solid background in future investigations for the determination of other properties such as tribological ones.

## 4. Conclusions

Conscious and skillful shaping of the properties of materials yields a number of benefits, such as the possibility of implementing improvements that have financial, safety and environmental benefits by reducing waste production at the design or adaptation stages [41,42,43,44,45]. Undoubtedly, there are helpful predictive models, and their appropriate use gives a view of how the modifications affect the properties of the manufactured material. Deep learning has been gaining popularity unabatedly in the area of data mining for some time now. Deep learning applies to multilayer neural networks, which act both as a generator of diagnostic features for the process under analysis and as a final classifier or regression system. Moreover, this approach to the non-interventional feature generation method is much more efficient than the traditionally used descriptor generation methods. At the same time, it makes it possible to improve the accuracy of the system. For this reason, deep network technology has recently and very quickly become one of the most popular areas in computer science.

By combining expert knowledge, research results obtained in empirical studies, as well as by using the Python programming language and available libraries, a neural network generating the predicted values of selected properties of polymer composites differing in composition (different content of alundum of various grain sizes as reinforcement) was proposed. By teaching and testing the network and then the obtained determination coefficient values, the optimal MLP structure for the case was proposed.

The comparison of forecast values with the values obtained at the stage of laboratory tests confirmed the effectiveness of the network (63% of forecasts classified as very accurate, 15% of forecasts defined as accurate).

The results encourage experimental trials for compositions that exhibit improved properties according to the generated predictions. It is also advisable to carry out simulations for other properties determined for the produced powder composites with the addition of alumina, as well as to determine the validity of its use after the introduction of fabrics into the composition of composites (hybrid composites).

Further modifications to the structure of the proposed network are also possible, which could generate more accurate forecasts. Operating on the basis of the Python programming language gave the freedom and flexibility while shaping the character of the program generating the predicted values of mechanical properties of composites. However, it is important in this case to rely on expertise in both decision making when selecting predictive properties and that in data science.

## Figures and Tables

**Table 1:** MSE values obtained for five types of model architectures.

	Mse Values For Individual Data Sets			
Model	Hardness	E_t_	σ_m_	ε_m_
Decision Tree Regressor	1.5596	6057.3994	26.0022	0.1032
MLP Regressor	1.4614	6259.9139	25.2744	0.1060
Linear Regression	1.4810	10761.3150	50.4864	0.1851
SVR	1.4686	25829.4423	48.8113	0.1326
K-Neighbors Regressor	1.5443	7146.3127	31.3305	0.1198

**Table 2:** Selected results of the coefficient of determination.

	Activation	Alpha	Hidden Layer Sizes	R^2^ Score
0	logistic	0.0001	23	0.329345321
1	logistic	0.0001	25	0.316109079
2	logistic	0.0001	19	0.313990104
3	logistic	0.001	25	0.312857918
4	logistic	0.0001	21	0.293111995
5	logistic	0.001	21	0.285338460
6	logistic	0.0001	17	0.269040464
7	logistic	0.001	19	0.263106927
8	logistic	0.001	23	0.242614750
9	identity	0.0001	23	0.229048600
10	identity	0.0001	13	0.226608287
11	identity	0.0001	9	0.225083033
12	logistic	0.001	17	0.223577093
13	identity	0.001	7	0.218035216
14	identity	0.001	5	0.212440871
15	identity	0.0001	25	0.203615714
16	identity	0.001	17	0.202399900
17	identity	0.001	19	0.198601455
18	identity	0.0001	17	0.196738083
19	identity	0.0001	5	0.196004000
20	identity	0.0001	7	0.188281408

**Table 3:** ME and MPE error values derived from prediction values for E_t_, σ_m_ and ε_m_.

	E_t_		σ_m_		ε_m_		Hardness	
Composition of The Composite	ME	MPE [%]	ME	MPE [%]	ME	MPE [%]	ME	MPE [%]
EA 220/5	13.93	0.60%	1.97	4.09%	0.11	4.90%	−0.55	−0.68%
EA 220/10	41.40	1.84%	3.46	6.84%	0.16	6.63%	0.33	0.39%
EA 220/15	−11.82	−0.49%	−1.37	−3.20%	−0.08	−4.10%	−1.88	−2.32%
EA 220/20	−8.40	−0.32%	2.08	4.46%	0.26	12.29%	−0.36	−0.45%
EA 220/25	−23.13	−0.84%	0.19	0.40%	−0.01	−0.40%	0.28	0.34%
EA 240/5	−21.78	−0.91%	0.90	1.52%	0.21	7.73%	1.18	1.43%
EA 240/10	9.72	0.39%	2.81	4.82%	0.16	5.82%	−0.10	−0.12%
EA 240/15	21.17	0.83%	2.56	4.78%	0.16	6.46%	0.03	0.03%
EA 240/20	−8.19	−0.31%	3.17	6.31%	0.17	8.23%	0.52	0.62%
EA 240/25	−37.17	−1.44%	−2.27	−5.92%	−0.03	−2.05%	−0.05	−0.06%
EA 280/5	33.63	1.34%	−1.12	−2.29%	−0.14	−7.04%	−0.68	−0.83%
EA 280/10	−92.36	−3.88%	−1.82	−3.65%	−0.05	−2.15%	−0.13	−0.15%
EA 280/15	38.43	1.53%	−3.05	−6.28%	−0.26	−12.56%	−0.20	−0.24%
EA 280/20	24.49	0.97%	0.72	1.70%	0.03	1.95%	0.19	0.23%
EA 280/25	−1.65	−0.06%	−2.29	−5.63%	−0.08	−5.24%	−0.12	−0.15%
EA 320/5	−14.67	−0.63%	2.11	3.98%	0.11	4.62%	0.69	0.82%
EA 320/10	−12.92	−0.52%	−0.04	−0.07%	0.00	−0.05%	0.25	0.29%
EA 320/15	2.56	0.10%	−1.48	−2.62%	−0.15	−6.46%	−0.19	−0.22%
EA 320/20	−47.68	−1.83%	−2.42	−4.33%	−0.15	−6.36%	0.09	0.11%
EA 320/25	−21.90	−0.84%	−2.30	−6.23%	−0.07	−5.18%	0.20	0.24%
EA 360/5	9.27	0.40%	−3.91	−8.40%	−0.20	−9.15%	0.74	0.89%
EA 360/10	0.99	0.04%	1.94	3.11%	0.10	3.37%	−0.31	−0.37%
EA 360/15	66.16	2.70%	0.90	1.45%	0.04	1.43%	−0.05	−0.07%
EA 360/20	−10.27	−0.40%	−0.45	−0.84%	−0.08	−3.36%	−0.70	−0.85%
EA 360/25	−29.81	−1.07%	3.97	7.65%	−0.13	−6.25%	−0.33	−0.39%

## Data Availability

Not applicable.
